# The incidence and risk factors of sepsis following ovarian cancer surgery: A retrospective Nationwide Inpatient Sample database study

**DOI:** 10.1371/journal.pone.0353675

**Published:** 2026-07-20

**Authors:** Yu Wang, Jing Zheng, Qun Lv, Guang-Min Xu, Hao Xie, Bo-Fei Dong, Xue Wang

**Affiliations:** 1 Department of Reproductive Medicine Center, Sichuan Provincial People’s Hospital, University of Electronic Science and Technology of China, Chengdu, China; 2 Department of Anesthesia and Surgery Center, Sichuan Provincial People’s Hospital, University of Electronic Science and Technology of China, Chengdu, China; 3 Division of Orthopaedic Surgery, Department of Orthopaedics, Nanfang Hospital, Southern Medical University, Guangzhou, Guangdong, China; 4 The Second School of Clinical Medicine, Southern Medical University, Guangzhou, Guangdong, China; University Hospitals of Cleveland: University Hospitals, UNITED STATES OF AMERICA

## Abstract

**Background:**

Postoperative sepsis is a significant complication following ovarian cancer surgery. However, limited studies have explored the risk factors associated with postoperative sepsis following ovarian cancer surgery in this context. This study aimed to assess the prevalence of postoperative sepsis and identify its associated risk factors.

**Methods:**

This study retrospectively analyzed data from patients who underwent ovarian cancer surgery between January 2010 and December 2019 using the Nationwide Inpatient Sample (NIS) database. The age range of the study population is between 18 and 99 years old. Patients were categorized into two groups based on the presence or absence of postoperative sepsis. Data on patient demographics (e.g., race, age), hospital characteristics (e.g., insurance type, bed size, teaching status, region), preoperative comorbidities, and postoperative complications were extracted for comparison. Univariate and multivariable logistic regression analysis were conducted to identify factors associated with postoperative sepsis.

**Results:**

A total of 39,049 patients were identified in the NIS database. Among them, 1,288 cases of postoperative sepsis were observed, representing an incidence rate of 3.3%. Patients with postoperative sepsis exhibited higher hospital charges, advanced age, prolonged length of stay (LOS), and increased in-hospital mortality. Preoperative risk factors for postoperative sepsis included congestive heart failure, coagulopathy, metastatic cancer, etc. Postoperative sepsis was associated with major complications, including electrolyte imbalance, urinary tract infection, thrombocytopenia, etc.

**Conclusions:**

The incidence of postoperative sepsis following ovarian cancer surgery has shown a slight increase over time. Postoperative sepsis is associated with advanced age, race, prolonged LOS, higher hospital charges, in-hospital mortality, preoperative comorbidities, and perioperative complications. Recognizing these risk factors is essential for improving patient prognosis.

## Introduction

Ovarian cancer is the third most common gynecological malignancy worldwide [1] and it is the most lethal female reproductive malignant tumor [2]. Due to the absence of early symptoms and effective screening methods, most patients are diagnosed at stage III or IV of the disease, with a 5-year survival rate of less than 30% [3]. Women suspected of having ovarian cancer generally undergo surgery to confirm the diagnosis, assess the extent of disease spread (surgical staging), or remove the tumor, either partially or completely [4]. Previous research has demonstrated that survival outcomes are strongly correlated with the size of residual tumor mass, with the greatest benefits observed when the tumor is completely removed [5]. Consequently, surgeons often strive to excise as many visible lesions as possible. Expanded cytoreduction (ECR) surgery, recommended by the National Comprehensive Cancer Network (NCCN) [6], aims for complete cytoreduction. Nathaniel et al. observed an increase in the number of extensive surgeries performed on women with ovarian cancer [7]. However, highly complex procedures elevate the risk of serious postoperative complications, such as sepsis [8]. Additionally, ovarian cancer patients are frequently elderly and have multiple comorbidities, predisposing them to severe postoperative complications, with sepsis being one of the most critical [9].

Sepsis is a leading cause of inpatient mortality, with surgical patients accounting for approximately one-third of all sepsis cases [10]. It is caused by a severe infection stemming from pathogenic microbes originating in any part of the body [11]. Immunosuppression significantly increases the risk of sepsis by reducing the bodys ability to fight infections [12]. Studies using animal models have shown that local malignancies can induce systemic immune alterations, increasing susceptibility to distant infections [13]. Epidemiological studies indicate that sepsis is 10 times more common in cancer patients than in individuals without a history of cancer [14]. Moreover, research has found that while the incidence of severe sepsis is lower in women than in men, the mortality rate for women in intensive care units (ICUs) is higher [15]. Given these characteristics of ovarian cancer patients, early prevention of postoperative sepsis is crucial for improving patient prognosis.

Despite its significance, there is currently a lack of research on the risk factors of postoperative sepsis following ovarian cancer surgery. To the best of our knowledge, this is the first study to explore the risk factors of sepsis in this specific patient population. Therefore, this study aimed to assess the prevalence of postoperative sepsis and identify its associated risk factors using data from the National Inpatient Sample (NIS). The findings are intended to guide clinical efforts in preventing postoperative sepsis effectively.

## Materials and methods

### Data source and ethical approval statement

The NIS database is the largest all-payer inpatient healthcare public database in the United States [16] It annually includes a stratified sample of 20% of hospitalizations from over 1,000 United States of America hospitals in 44 states participating in the Healthcare Cost and Utilization Project (HCUP) [17]. Data for this study were derived from this database. As the NIS data are de-identified and publicly accessible, this study was exempt from informed consent and Institutional Review Board (IRB) approval. The study strictly adheres to the Declaration of Helsinki and STROBE reporting guidelines.

### Study population

This study retrospectively analyzed data from patients who underwent ovarian cancer surgery between January 2010 and December 2019, as recorded in the NIS database. The study population was identified using International Classification of Diseases, Ninth and Tenth Revisions, Clinical Modification (ICD-9-CM/ICD-10-CM) procedure codes (S1 Table) for ovarian cancer, ovarian cancer surgery, and sepsis. Ovarian cancer surgery included removal of uterus, fallopian tubes, ovaries, and lymph nodes, as well as ECR surgery such as removal of the intestine, liver, and spleen.

**Fig 1:**
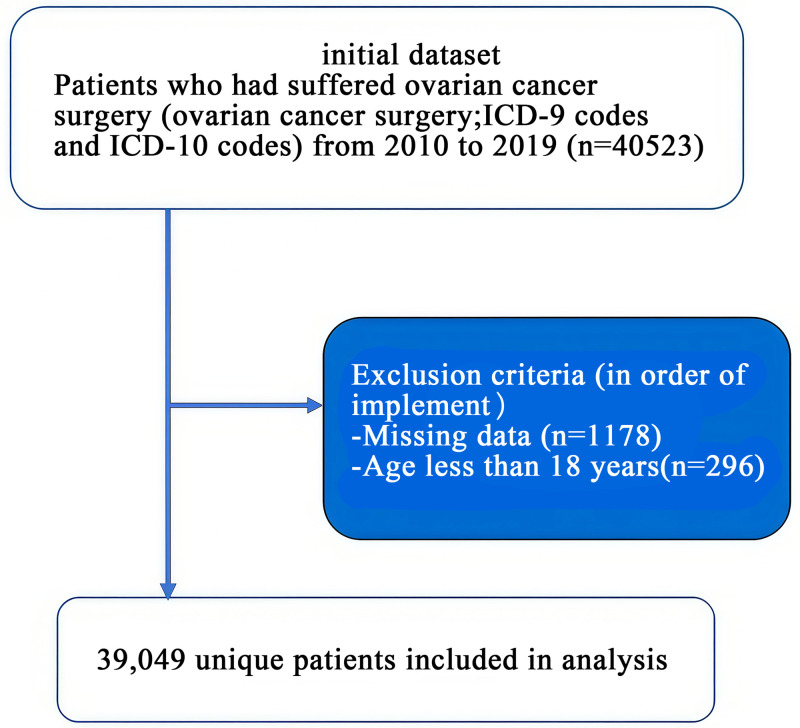
Exclusion process for patients with ovarian cancer surgery.

A total of 40,227 unique patients aged 18 years or older were initially identified. After excluding patients with missing data—including age (4), gender (28), total charges (805), mortality status (15), insurance type (54), hospital bed size (133), and elective admission status (139)—39,049 patients were included in the final analysis (Fig 1). This missingness was random and the number of patients with missing data was only 2.93% of the total number of cases. So, according to previous literature, the impact of the missingness is likely to be small [18]. Patients were categorized into two groups based on whether they developed sepsis after surgery. Within these groups, patients were stratified by age into the following subgroups: 18–44, 45–64, 65–74, and≥75 years.

### Covariates and study variables

Data on patient demographics, hospital characteristics, preoperative comorbidities, and postoperative complications were extracted for analysis (Table 1). Patient demographics included age group, race, number of comorbidity, and type of insurance. Hospital characteristics included bed size of hospital, elective admission, teaching status of hospital, region of hospital, and location of hospital.

**Table 1:** Variables used in binary logistic regression analysis.

Variables Categories	Specific Variables
Patient demographics	Age (≥18 years), race (White, Black, Hispanic, Asian or Pacific Islander, Native American and Other), type of insurance (Medicare, Medicaid, private insurance, self-pay, no charge, other)
Hospital characteristics	Type of admission (non-elective, elective), bed size of hospital (small, medium, large), teaching status of hospital (nonteaching, teaching), location of hospital (rural, urban), region of hospital (northeast, Midwest or north central, south, west)
Comorbidities	deficiency anemia, rheumatoid arthritis/collagen vascular diseases, chronic blood loss anemia, congestive heart failure, chronic pulmonary disease, coagulopathy, depression, diabetes (uncomplicated), diabetes (with or without chronic complications), hypertension (combine uncomplicated and complicated), hypothyroidism, liver disease, fluid and electrolyte disorders, metastatic cancer, other neurological disorders, obesity, peripheral vascular disorders, psychoses, pulmonary circulation disorders, renal failure, solid tumor without metastasis, valvular disease and weight loss
complications	electrolyte imbalance, ileus, blood transfusion, urinary tract infection, thrombocytopenia, respiratory disease, genitourinary disease, gastrointestinal complication, pulmonary embolism, respiratory failure, continuous mechanical ventilation in trauma, heart failure, deep vein thrombosis, pneumonia, urinary retention, posthemorrhagic anemia, wound infection

AIDS: Acquired immunodeficiency syndrome.

Preoperative comorbidities and postoperative complications are presented in Table 1. The selection of preoperative comorbidities and postoperative complications was based on a review of existing publications [8,19,20]and the authors’ expertise. ICD-9-CM and ICD-10-CM diagnosis codes were used to extract information on complications from the database.

Since there is no chronological record of the occurrence of various postoperative complications in the NIS database, the causal relationship between sepsis and other complications cannot be clarified, so we did not include postoperative complications-related variables when building the risk prediction model.Variables of the risk factor prediction model included age, race, the number of comorbidities, hospital location, hospital region, insurance type, bed size, elective admission, teaching status of hospital, congestive heart failure, pulmonary circulation disorders, diabetes with chronic complications, valvular disease, metastatic cancer, hypothyroidism, fluid and electrolyte disorders, coagulopathy, hypertension, solid tumor without metastasis, rheumatoid arthritis, peripheral vascular disorders, other neurological disorders, and weight loss.

### Model development

In order to avoid over-adjustment, we used a Directed Acyclic Graph (DAG) to select variables from patient demographics, hospital characteristics and preoperative comorbidities for multivariable logistic regression analysis to construct the risk factor prediction model (S1 Fig.). First, all potential variables associated with the exposure-outcome relationship were screened and enumerated based on pathophysiological plausibility, clinical relevance, epidemiological evidence, and prior published literature. These potential variables were subsequently used to establish a fully saturated initial causal DAG network. Guided by the constructed DAG, we identified a sufficient adjustment set to estimate the total causal effect. Specifically, variables acting as mediators or colliders were explicitly excluded from the regression model to prevent overadjustment and collider bias, respectively. By adjusting solely for the necessary confounders, all open backdoor paths were completely blocked to eliminate confounding bias. Variables with extremely low incidence were further excluded from the reserved variables to prevent unstable fitting and overfitting of the model caused by insufficient effective sample size. Multicollinearity diagnosis was conducted via the variance inflation factor (VIF). Redundant variables with highly overlapping clinical information were eliminated, and only the most clinically representative indicator was retained within each collinear variable group.

DAGs are graphical tools that visualize causal relationships between variables, typically constructed based on theoretical frameworks or existing research [21]. In a DAG, nodes represent variables, and directed edges (single-headed arrows) indicate the hypothesized direction of causal effects. Because DAGs assume no feedback loops (meaning a variable cannot be its own cause, directly or indirectly), the graph is acyclic [21]. In this study, the DAG was constructed to identify confounding variables and guide appropriate covariate selection for inclusion in the regression model.

In multiple regression, multicollinearity refers to high linear correlations among independent variables [22]. When present, multicollinearity makes it difficult to isolate the independent contribution of each predictor, resulting in unstable coefficient estimates and substantially inflated standard errors, thereby reducing the reliability of statistical inference [22]. The Variance Inflation Factor (VIF) is a standard diagnostic measure for multicollinearity. In this study, a conservative threshold of VIF > 5 was applied, and redundant predictors were removed to reduce the VIF to acceptable levels.

Prior to constructing the multivariable logistic regression model, rare categorical covariates with sparse strata were excluded from the model. In logistic regression, sparse data can lead to complete or quasi-complete separation [23]. Under such conditions, maximum likelihood estimates fail to converge, yielding infinite or undefined coefficient estimates, severely inflated standard errors, and implausible odds ratio estimates [24]. By excluding these rare independent variables, we prevented spurious multicollinearity, preserved statistical power, and reduced the risk of model overfitting.

### Statistical analysis

Data analysis was conducted using SPSS statistical software version 25.0. Statistical significance was set at P < 0.05. Continuous variables were summarized as medians with interquartile ranges (P25, P75) and categorical variables as frequencies with percentages. We used the Kolmogorov-Smirnov test to assess the normality of continuous variables. As all continuous variables in this study did not conform to a normal distribution, the Wilcoxon rank-sum test was used for univariate analysis. Categorical variables were analyzed using the chi-square test. Multivariable logistic regression analysis was performed to identify which variables in patient demographic characteristics, hospital characteristics, preoperative comorbidities, and postoperative complications were risk factors for postoperative sepsis, with results presented as odds ratios (ORs) and 95% confidence intervals (CIs). To avoid multicollinearity, variables with vif greater than 5 are excluded from the model and comorbidities with an incidence rate of less than 1% were excluded from the model. The performance of model were checked with area under the curve (AUC).

## Results

### Incidence of sepsis in patients undergoing ovarian cancer surgery

**Fig 2:**
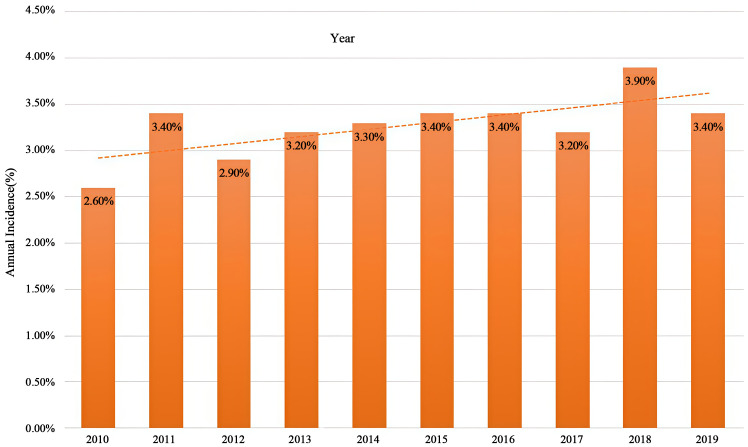
Annual incidence of sepsis after ovarian cancer surgery.

A total of 39,049 patients who underwent ovarian cancer surgery were analyzed from the NIS database between 2010 and 2019 in the United States. Among them, there were 1,288 cases of postoperative sepsis, with an overall incidence rate of 3.3% (1,288/39,049) (Table 2). The trend in postoperative sepsis rates fluctuated over the years: from 2.6% (117/4,510) in 2010 to 3.4% (137/4,057) in 2011, followed by a decrease to 2.9% (117/4,037) in 2012 (Fig 2). Subsequently, the annual incidence rates steadily increased from 2012 to 2018 (rising from 2.9% (117/4,037) to 3.9% (143/3,707)), before slightly declining to 3.4% (126/3,744) in 2019. However, the rate in 2019 remained higher than that observed in 2012 (Fig 2).

**Table 2:** Patient characteristics and outcomes after ovarian cancer surgery (2010-2019).

Characteristics	sepsis	No sepsis	P
Total (n = count)	1,288	37,761	
Total incidence (%)	3.30		
Age (median, years)	66 (56, 74)	62(52, 70)	<0.001*
Age group (%)			
18-44	7.2	11.1	<0.001*
45-64	38.9	46.8	
65-74	29.3	26.5	
≥ 75	24.6	15.6	
Race (%)			
White	66.9	71.6	<0.001*
Black	10.2	7.3	
Hispanic	9.0	7.7	
Asian or Pacific Islander	3.3	3.6	
Native American	0.6	0.4	
Other	9.9	9.3	
Number of Comorbidity (%)			
0	1.2	12.3	<0.001*
1	5.7	21.1	
2	10.7	22.9	
≥ 3	82.5	43.7	
LOS (median, d)	17 (11-27)	5 (3-8)	<0.001*
TOTCHG (median, $)	192,469.5 (111,106.75-324,302.75)	60,548 (39,741−95,481.5)	<0.001*
Type of insure (%)			
Medicare	53.7	40.5	<0.001*
Medicaid	10.2	8.6	
Private insurance	31.5	44.8	
Self-pay	2.6	3.2	
No charge	0.3	0.4	
Other	1.7	2.4	
Bed size of hospital (%)			
Small	8.7	8.4	0.138
Medium	23.8	21.6	
Large	67.5	69.9	
Elective admission (%)	36.6	79.4	<0.001*
Type of hospital (teaching %)	76.1	82.7	<0.001*
Location of hospital (urban, %)	96.5	97.9	0.001*
Region of hospital (%)			
Northeast	16.6	20.5	0.001*
Midwest or North Central	21.4	22.5	
South	39.8	35.4	
West	22.1	21.6	
In-hospital mortality (%)	21.7	0.7	<0.001*

LOS: Length of stay; TOTCHG: Total charge.

*These *p* values indicate statistical significance.

### Patient demographics between the two groups

Patients with postoperative sepsis were significantly older than those without sepsis. The median age was significantly higher in the sepsis group compared to the non-sepsis group (66 years [IQR 56–74] vs. 62 years [IQR 52–70], *P* < 0.001). Furthermore, age distribution differed significantly between the groups, with a 9.0% higher incidence of postoperative sepsis in patients older than 75 years (*P* < 0.001) (Table 2). There were also differences in racial distribution between the two groups (*P* < 0.001). The incidence of postoperative sepsis was lower among White (66.9% (862/1,288) vs. 71.6% (27053/37,761)) and Asian or Pacific Islander (3.3% (42/1,288) vs. 3.6% (1,375/37,761)) patients compared to those without sepsis (*P* < 0.001).The incidence of postoperative sepsis was higher among Black (10.2% (131/1,288) vs. 7.3% (2757/37,761)), Hispanic (9.0% (116/1,288) vs. 7.7% (2908/37,761)), Native American (0.6% (77/1,288) vs. 0.4% (1,510/37,761)), and other race(9.6% (124/1,288) vs. 9.3% (3511/37,761)) patients compared to those without sepsis (*P* < 0.001) (Table 2).

### Hospital characteristics between the two groups

Regarding insurance coverage, government insurance (Medicare and Medicaid) was the primary type for patients with sepsis (53.7% (692/1,288) and 10.2% (131/1,288), respectively), followed by private insurance (31.5% (406/1,288)) and other types of coverage (*P* < 0.001) (Table 2). However, no significant difference was observed regarding hospital bed size (Table 2). Elective admissions were less common in the sepsis group (36.6% (471/1,288) vs. 79.4% (29,985/37,761); *P* < 0.001) (Table 2). Additionally, teaching hospitals showed a lower incidence of postoperative sepsis (76.1% (980/1,288) vs. 82.7% (31,223/37,761); *P* < 0.001). Urban hospitals also were less common in the sepsis group (96.5% (1,243/1,288) vs. 97.9% (36,972/37,761); *P* = 0.001) (Table 2). Geographical differences were also significant. There is a difference between the distribution of the the region of hospital in patients with sepsis and those without sepsis. (*P* = 0.001) (Table 2).

### Adverse impact of postoperative sepsis after ovarian cancer surgery

There is a difference between the distribution of the number of comorbidities in patients with sepsis and those without sepsis. Patients with sepsis had significantly more comorbidities, with 82.5% (1,062/1,288) having a number of comorbidities ≥3 compared to 43.7% (16,499/37,761) in the non-sepsis group (Table 2). The median length of stay (LOS) for sepsis patients was extended by 12 days compared to those without sepsis (11–27 days vs. 3–8 days; *P* < 0.001). Prolonged stays and higher costs were considered only when the length of stay (LOS) and total cost exceeded 75.00%, respectively. Additionally, the median total hospitalization charges were significantly higher for sepsis patients, with an increase of $131,921.50 ($192,469.50 vs. $60,548; *P* < 0.001) (Table 2). The mortality rate among patients with postoperative sepsis was more than 31 times higher than that of non-sepsis patients (21.7% (279/1,288) vs. 0.7% (248/37,761); *P* < 0.001) (Table 2).

### Relationship between sepsis and preoperative comorbidities

Patients with certain preoperative comorbidities, including deficiency anemia (17.9% (231/1,288)), chronic blood loss anemia (3.2%(41/1,288)), congestive heart failure (11.3%(146/1,288)), coagulopathy (21.8%(281/1,288)), diabetes with chronic complications (6.4%(82/1,288)), liver disease (4.0%(52/1,288)), fluid and electrolyte disorders (68.0%(876/1,288)), metastatic cancer (53.0%(683/1,288)), and weight loss (42.2%(543/1,288)) (*P* < 0.001) were more common among patients with sepsis. Additional comorbidities associated with sepsis included rheumatoid arthritis/collagen vascular diseases (1.2% (15/1,288); *P* = 0.027), chronic pulmonary disease (14.1% (182/1,288); *P* = 0.004), drug abuse (1.2% (15/1,288); *P* = 0.041), hypertension (46.7% (601/1,288); *P* = 0.020), obesity (16.4% (211/1,288);*P* = 0.024),and psychoses (2.9% (37/1,288); *P* = 0.010) (Table 3).

**Table 3:** Relationship between sepsis and preoperative comorbidities.

Comorbidities	Univariate Analysis		
	No sepsis	sepsis	P
Preoperative comorbidities			
Deficiency anemia	4,426 (11.70%)	231 (17.90%)	<0.001*
Rheumatoid arthritis/collagen vascular diseases	773 (2.00%)	15 (1.20%)	0.027*
Chronic blood loss anemia	640 (1.70%)	41 (3.20%)	<0.001*
Congestive heart failure	949 (2.50%)	146 (11.30%)	<0.001*
Chronic pulmonary disease	4,350 (11.50%)	182 (14.10%)	0.004*
Coagulopathy	1,411 (3.70%)	281 (21.80%)	<0.001*
Depression	4,134 (10.90%)	138(10.70%)	0.792
Diabetes, uncomplicated	4,230 (11.20%)	150 (11.60%)	0.620
Diabetes with chronic complications	931(2.50%)	82(6.40%)	<0.001*
Hypertension	16,382 (43.40%)	601 (46.70%)	0.020*
Hypothyroidism	5,748 (15.20%)	176(13.70%)	0.125
Liver disease	851 (2.30%)	52 (4.00%)	<0.001*
Fluid and electrolyte disorders	7,845 (20.80%)	876 (68.00%)	<0.001*
Metastatic cancer	11,530 (30.50%)	683(53.00%)	<0.001*
Other neurological disorders	954(2.50%)	123 (9.50%)	<0.001*
Obesity	5,344 (14.20%)	211 (16.40%)	0.024*
Peripheral vascular disorders	766 (2.00%)	76 (5.90%)	<0.001*
Psychoses	709 (1.90%)	37 (2.90%)	0.010*
Pulmonary circulation disorders	779 (2.10%)	97 (7.50%)	<0.001*
Renal failure	1,251 (3.30%)	114 (8.90%)	<0.001*
Solid tumor without metastasis	13,708 (36.30%)	610 (47.40%)	<0.001*
Valvular disease	1,063 (2.80%)	40(3.10%)	0.536
Weight loss	3,048 (8.10%)	543 (42.20%)	<0.001*

*These *p* values indicate statistical significance.

### Risk factors related to postoperative sepsis after ovarian cancer surgery

Risk factors for postoperative sepsis were identified through multivariable logistic regression analysis. Race was a significant factor, with “Hispanic” racial groups showing increased risk (OR = 1.26; CI = 1.01–1.57; *P* = 0.045). The number of comorbidities was also strongly associated, with patients having ≥3 comorbidities showing the highest risk (OR = 5.32; CI = 3.12–9.08; *P* < 0.001). Logistic regression analysis further identified significant findings, congestive heart failure (OR=2.26; CI = 1.82–2.80), coagulopathy (OR=2.92; CI = 2.48–3.44), diabetes with chronic complications (OR=1.53; CI = 1.17–1.98), fluid and electrolyte disorders (OR=3.07;CI = 2.67–3.52), other neurological disorders (OR=2.00;CI = 1.60–2.50), peripheral vascular disorders (OR=1.67;CI = 1.26–2.20), pulmonary circulation disorders (OR=1.71;CI = 1.34–2.19), weight loss (OR=2.67;CI = 2.34–3.06) (Table 3).Conversely, protective factors included Self-pay (OR = 0.64; CI = 0.42–1.00; P = 0.035), elective admissions (OR = 0.31; CI = 0.27–0.35; P < 0.001) rheumatoid arthritis/collagen vascular diseases (odds ratio [OR]=0.46; 95% confidence interval [CI]=0.27–0.78; P = 0.004), hypertension (OR=0.65; CI = 0.57–0.74; *P* < 0.001), hypothyroidism (OR=0.76;CI = 0.64–0.91; P = 0.002) and valvular disease (OR=0.57;CI = 0.40–0.81; P = 0.002). (Table 4).

**Table 4:** Risk factors associated with sepsis after ovarian cancer surgery.

Variable	Multivariable Logistic Regression		
	OR	95% CI	P
Age(years)			
18-44	Ref		
45-64	1.16	0.91–1.47	0.246
65-74	1.06	0.79–1.44	0.685
≥ 75	1.15	0.83–1.57	0.401
Race			
White	Ref	——	——
Black	1.21	0.98–1.50	0.074
Hispanic	1.26	1.01–1.57	0.045*
Asian or Pacific Islander	1.01	0.72–1.43	0.935
Native American	1.85	0.84–4.05	0.126
Other	1.2	0.98–1.48	0.081
Number of Comorbidity			
0	Ref	——	——
1	2.37	1.36–4.15	0.002*
2	3.15	1.84–5.41	<0.001*
≥ 3	5.32	3.12–9.08	<0.001*
Type of insurance			
Medicare	Ref	——	——
Medicaid	0.93	0.71–1.22	0.604
Private insurance	0.82	0.66–1.00	0.052
Self-pay	0.64	0.42–1.00	0.035*
No charge	0.73	0.25–2.16	0.571
Other	0.69	0.43–1.12	0.135
Bed size of hospital			
Small	Ref	——	——
Medium	1.09	0.86–1.38	0.494
Large	1.04	0.84–1.29	0.705
Elective admission	0.31	0.27–0.35	<0.001*
Teaching hospital	0.89	0.76–1.05	0.165
Urban hospital	0.8	0.56–1.14	0.217
Region of hospital			
Northeast	Ref	——	——
Midwest or North Central	0.93	0.76–1.13	0.462
South	1.11	0.93–1.32	0.252
West	1.03	0.85–1.26	0.749
Rheumatoid arthritis/collagenvascular diseases	0.46	0.27–0.78	0.004*
Congestive heart failure	2.26	1.82–2.80	<0.001*
Coagulopathy	2.92	2.48–3.44	<0.001*
Diabetes with chronic	1.53	1.17–1.98	0.002*
Complications			
Hypertension	0.65	0.57–0.74	<0.001*
Hypothyroidism	0.76	0.64–0.91	0.002*
Fluid and electrolyte disorders	3.07	2.67–3.52	<0.001*
Metastatic cancer	1.00	0.86–1.16	0.95
Other neurological disorders	2.00	1.60–2.50	<0.001*
Peripheral vascular disorders	1.67	1.26–2.20	<0.001*
Pulmonary circulation disorders	1.71	1.34–2.19	<0.001*
Solid tumor without metastasis	0.91	0.79–1.06	0.233
Valvular disease	0.57	0.40–0.81	0.002*
Weight loss	2.67	2.34–3.06	<0.001*

OR: Odds ratio; CI: Confidence interval.

*These *p* values indicate statistical significance.

### Relationship between sepsis and postoperative complications

Patients with postoperative sepsis were more likely to experience complications such as electrolyte imbalance (59.6% (768/1,288)), ileus (26.2% (337/1,288)), blood transfusion (38.7% (498/1,288)), urinary tract infection (22.0% (284/1,288)), thrombocytopenia (10.3% (133/1,288)), respiratory disease(4.1% (53/1,288)), genitourinary disease(57.7% (743/1,288)), gastrointestinal complication(8.8% (113/1,288)), pulmonary embolism(7.8% (101/1,288)), continuous mechanical ventilation in trauma(22.9% (295/1,288)), heart failure(8.5% (109/1,288)), deep vein thrombosis(10.5% (135/1,288)), pneumonia(19.7% (254/1,288)), posthemorrhagic anemia(37.7% (485/1,288)), wound infection(14.6% (188/1,288)), and respiratory failure(17.3% (233/1,288))(*P* < 0.001) (Table 5).

**Table 5:** Relationship between sepsis and postoperative complications.

Complications	Univariate Analysis			Multivariable Logistic Regression		
	No sepsis	sepsis	P	OR	95% CI	P
Medical complications						
Electrolyte imbalance	6622 (17.50%)	768 (59.60%)	<0.001	2.82	2.47-3.22	<0.001*
Ileus	5832 (15.40%)	337 (26.20%)	<0.001	0.98	0.84-1.15	0.818
Blood transfusion	7452 (19.70%)	498 (38.70%)	<0.001	1.13	0.99-1.3	0.075
Urinary tract infection	2040 (5.40%)	284 (22.00%)	<0.001	2.0	1.65-2.45	<0.001*
Thrombocytopenia	893 (2.40%)	133 (10.30%)	<0.001	2.11	1.67-2.66	<0.001*
Respiratory disease	615 (1.60%)	53 (4.10%)	<0.001	0.75	0.53-1.06	0.101
Genitourinary disease	4497 (11.90%)	743 (57.70%)	<0.001	1.15	0.88-1.5	0.321
Gastrointestinal complication	1295(3.40%)	113(8.80%)	<0.001	1.96	1.53-2.51	<0.001*
Pulmonary embolism	721 (1.90%)	101(7.80%)	<0.001	1.87	1.43-2.45	<0.001*
Respiratory failure	769(2.00%)	233(17.30%)	<0.001	2.33	1.9-2.87	<0.001*
Continuous mechanical ventilation in trauma	637(1.70%)	295(22.90%)	<0.001	4.16	3.43-5.04	<0.001*
Heart failure	724(1.90%)	109(8.50%)	<0.001	1.5	1.16-1.95	0.002*
Deep vein thrombosis	792 (2.10%)	135 (10.50%)	<0.001	1.85	1.45-2.36	<0.001*
Pneumonia	854 (2.30%)	254 (19.70%)	<0.001	3.95	3.27-4.78	<0.001*
Urinary retention	986(2.60%)	29 (2.30%)	0.425	0.628	0.42-0.95	0.026*
Surgical complications						
Posthemorrhagic anemia	7890 (20.90%)	485 (37.70%)	<0.001	1.14	0.99-1.3	0.068
Wound infection	742(2.00%)	188(14.60%)	<0.001	5.02	4.08-6.19	<0.001*

OR: Odds ratio, CI: Confidence interval.

*These *p* values indicate statistical significance.

Multivariable analysis identified strong associations between sepsis and electrolyte imbalance (OR = 2.82; CI = 2.47–3.22), urinary tract infection (OR=2.0; CI = 1.65–2.45), thrombocytopenia (OR=2.11 CI = 1.67–2.66), gastrointestinal complication (OR=1.96; CI = 1.53–2.51), pulmonary embolism (OR=1.87; CI = 1.43–2.45), respiratory failure (OR=2.33; CI = 1.9–2.87), continuous mechanical ventilation in trauma (OR=4.16; CI = 3.43–5.04), heart failure (OR=1.5; CI = 1.16–1.95), deep vein thrombosis (OR=1.85; CI = 1.45–2.36), pneumonia (OR=3.95; CI = 3.27–4.78), urinary retention (OR=0.63; CI = 0.42–0.95), and wound infection (OR = 5.02; CI = 4.08–6.19) (Table 5). Among them, wound infection had the highest adjusted odds among sepsis patients, and it means that patients with sepsis had approximately five times the odds of experiencing postoperative wound infection. Additionally, urinary retention had the lowest adjusted odds among sepsis patients.

## Discussion

This study analyzes the risk factors, incidence, and health economics of postoperative sepsis in ovarian cancer patients. Our findings indicate an upward trend in the incidence of sepsis following ovarian cancer surgery from 2010 to 2019 (Fig 2). Compared to those without sepsis, patients who developed postoperative sepsis experienced significantly higher number of comorbidities, longer LOS, higher median total hospitalization charges, elevated mortality rates, and lower rates of private insurance, teaching hospitals, and urban hospitals. In addition, a variety of ovarian cancer postoperative complications are also associated with postoperative sepsis. Identifying and understanding the risk factors associated with postoperative sepsis is essential for its prevention.

Epidemiological studies suggest that Black patients are more likely to experience severe sepsis than White patients [25]. This may be due to higher infection rates and greater risks of acute organ dysfunction among Black individuals, although the underlying mechanisms remain unclear [26]. However, our studys multivariable logistic regression analysis identified “Hispanic” as independent risk factors for sepsis after ovarian cancer surgery, deviating from broader epidemiological trends. This discrepancy may stem from our studys focus on postoperative sepsis rather than severe sepsis. On the other hand, the incidence of sepsis was higher in men than in women, with the highest incidence among black men [27], and our study population was female patients, so this may be one of the reasons why the differences between blacks and whites in this study were not significant.

Socioeconomic status has also been extensively linked to health outcomes [28]. For example, Goodwin et al. reported that individuals residing in economically disadvantaged areas are more likely to suffer from severe sepsis [29]. Consistent with this, our research found that patients with self-pay, often indicative of higher income and better healthcare access, had a lower incidence of sepsis. So, expanding insurance coverage for low-income populations to improve their perioperative care is one measure to reduce the incidence of sepsis in these patients [30]. Additionally, previous study [20] and our findings show that elective admissions reduce the risk of postoperative complications. Elective cases typically involve healthier patients or those adequately prepared for surgery, whereas emergent cases are often more severe [31].

The pathology of sepsis involves dysregulation of the immune system in response to infection [32]. Patients with comorbidities that compromise immune function are at higher risk of sepsis, and the risk increases with the number of comorbidities. Rachel et al. identified bleeding disorders as predictors of sepsis in cervical laminectomy and fusion surgeries [33]. Similarly, our study identified coagulation dysfunction as an independent risk factor for postoperative sepsis, likely due to associated immunosuppressive conditions [33]. Diabetes has long been recognized as a sepsis risk factor. A United Kingdom cohort study found a higher prevalence of sepsis among individuals with diabetes compared to the general population [34]. Our findings corroborate this, with diabetes accompanied by chronic complications being associated with sepsis. Congestive heart failure, another identified risk factor, may increase sepsis susceptibility by impairing gut barrier function and enabling bacterial translocation [35]. Peripheral vascular diseases might also elevate sepsis risk by disrupting vascular integrity [36]. Fluid and electrolyte disorders emerged as significant risk factors for sepsis in our study, aligning with Luming et al.‘s findings [37]. Fluid imbalance can lead to organ hypoperfusion and subsequent organ failure, including gut barrier dysfunction. Additionally, fluid imbalance may impair leukocyte adhesion and neutrophil migration, exacerbating infection susceptibility [38]. Other neurological disorders were also identified as risk factors for sepsis. Emerging evidence suggested that gut microbiome alterations was a key factor in the development of neurological disorders [39]. Meanwhile, alterations in the gut microbiome increase susceptibility to sepsis through multiple mechanisms [40]. This connection warrants further investigation. Weight loss was also a risk factors for sepsis, likely due to weakened immune systems in these patients. Thus, if the patient has comorbidities that damage the bodys immune function or intestinal or vascular barrier functions, the comorbidities should be actively treated before the operation to reduce the risk of infection, such as restoring normal coagulation function, controlling blood sugar, improving heart function, correcting fluid and electrolyte disorders, and strengthening nutrition. Interestingly, hypertension was identified as a protective factor for postoperative sepsis. But evidence in human studies is limited, so the exact relationship between hypertension and sepsis remains unclear.

Our results also demonstrate that multiple postoperative complications of ovarian cancer surgery—including electrolyte imbalance, urinary tract infections, thrombocytopenia, gastrointestinal complications, pulmonary embolism, respiratory failure, mechanical ventilation, heart failure, deep vein thrombosis, pneumonia, and wound infections—are strongly associated with sepsis.

Infection-related complications, such as pneumonia, continuous mechanical ventilation in trauma, urinary tract infections, and wound infections, were prominent sepsis risk factors. Pneumonia, in particular, has been identified as the most common cause of sepsis [41]. Respiratory failure-associated sepsis may result from primary pulmonary infections or systemic inflammatory responses [42]. Patients with respiratory failure requiring mechanical ventilation are at heightened sepsis risk. So, in order to prevent the occurrence of postoperative sepsis after ovarian cancer surgery, doctors need to take effective measures to prevent and treat infection-related postoperative complications, such as early removal of urinary catheters, intermittent catheterization and rational use of antibiotics [43].

Thrombosis and coagulation dysfunction are also common in sepsis [44]. Consistent with this, our study found associations between sepsis and pulmonary embolism, deep vein thrombosis, and thrombocytopenia. In a model of photochemical injury-induced thrombosis, it was found that endotoxemia caused platelet adhesion in the microvessels, which in turn promotes intravascular thrombosis through vascular hemophilic factor (VWF), endotoxin signaling receptor TLR4, coagulation cascade reaction, inflammatory factor storm, and induction of fibrinolysis system degradation [44]. A greater than 55% of patients with sepsis have thrombocytopenia [45]. Thrombocytopenia may be a cause or a consequence of sepsis. As a result of host immune dysfunction in severely thrombocytopenic patients, leukocyte adhesion is reduced and complement signaling is enhanced, leading to an increased risk of sepsis [46]. In the immune response of sepsis, platelets are irreversibly expended, leading to thrombocytopenia [45]. Therefore, prevention and treatment of postoperative thrombosis may reduce the incidence and severity of sepsis. Clinicians should regularly assess the patients thrombosis risk during the hospitalization period, and use drugs such as low molecular weight heparins and mechanical treatment [47].

Lastly, our study found that patients with sepsis had a higher incidence of heart failure. Sepsis-induced cardiomyopathy (SICM) contributes to heart failure in sepsis patients, likely driven by microbial endotoxins, cytokines, and nitric oxide [48].

### Limitations

This study has several inherent limitations. First, its retrospective design may introduce selection bias. Second, the temporal relationship between postoperative sepsis and other complications (including pneumonia, deep vein thrombosis, and respiratory failure) could be bidirectional, and our database lacks the granularity to determine the precise sequence of these events. Third, the NIS database lacks follow-up data after discharge, which limits longitudinal analysis. Additionally, key variables such as cancer stage, tumor burden, extent of cytoreduction, surgery duration, perioperative medication use, and anesthesia recovery details were unavailable. Studies showed ovarian cancer severity and extent of surgery strongly associated with postoperative complications [49,50].The database also lacks standardized metrics for grading the severity of both comorbidities and complications. Furthermore, there is an inherent risk of misclassification due to the reliance on administrative coding rather than clinical documentation. A systematic review and meta-analysis showed that compared with chart review and registration, sepsis ICD-10 codes had lower positive predictive value (median, 72.0%; IQR, 50.0%−84.7%) and sensitivity (median, 41.9%; IQR, 19.3–57.5%) and higher negative predictive value (median, 95.9%; IQR, 85.5–98.3%) and specificity (median, 99.5%; IQR, 96.2–99.6%) [51]. Several studies showed comparable sensitivity values of ICD-9 and ICD-10 codes in selected situations [52,53]. These show that sepsis is undercoded in the administrative database, resulting in misclassification. Finally, the observational nature of this study prevents us from establishing definitive causal relationships between variables. To address these limitations and validate our findings, future prospective studies with detailed clinical data collection, standardized severity assessments, and longer follow-up periods are necessary.

## Conclusions

The incidence of postoperative sepsis increased annually, rising from 2.6% in 2010 to 3.9% in 2018, before declining slightly to 3.4% in 2019. A slight increase in overall morbidity risk was observed. The occurrence of sepsis following ovarian cancer surgery was associated with race, elective admission status, type of insurance, and the number of comorbidities. Postoperative sepsis was linked to increased hospital costs and longer durations of hospital stays. It was also significantly associated with various comorbidities and complications, including congestive heart failure, coagulopathy, diabetes with chronic complications, fluid and electrolyte disorders, metastatic cancer, other neurological disorders, peripheral vascular disorders, pulmonary circulation disorders, solid tumors without metastasis, weight loss, urinary tract infections, pulmonary embolism, and respiratory failure. Understanding the risk factors associated with postoperative sepsis is essential for improving adverse outcomes and ensuring effective management strategies.

## Supporting information

S1 Fig Directed Acyclic Graph (DAG). (DOCX)

S1 Table ICD-9-CM/ICD-10-CM procedure codes for ovarian cancer, ovarian cancer surgery, and sepsis. (DOCX)

S2 Table Definition of Bed size of hospital in the NIS database. NIS: National Inpatient Sample.
(DOCX)

S3 Table The ICD-9/10 code of complications. (DOCX)

## References

[1] Bray F, Ferlay J, Soerjomataram I, Siegel RL, Torre LA, Jemal A. Global cancer statistics 2018: GLOBOCAN estimates of incidence and mortality worldwide for 36 cancers in 185 countries. CA: A Cancer J Clin. 2018;68(6):394–424. 10.3322/caac.21492 30207593

[2] Wang L, Wang X, Zhu X, Zhong L, Jiang Q, Wang Y, et al. Drug resistance in ovarian cancer: from mechanism to clinical trial. Mol Cancer. 2024;23(1):66. doi: 10.1186/s12943-024-01967-3 38539161 PMC10976737

[3] NIH Consensus Conference, NIH Consensus Development Panel on Ovarian Cancer. Ovarian cancer. Screening, treatment, and follow-up. JAMA. 1995;273(6):491–7. 7837369

[4] Dodge JE. Epithelial ovarian cancer surgical staging by Ontario gynaecologic surgeons: is there a gap between current practice and the Canadian clinical practice guidelines? J Obstet Gynaecol Can. 2007;29(8):653–63. doi: 10.1016/s1701-2163(16)32550-6 17714619

[5] Chang S-J, Hodeib M, Chang J, Bristow RE. Survival impact of complete cytoreduction to no gross residual disease for advanced-stage ovarian cancer: a meta-analysis. Gynecol Oncol. 2013;130(3):493–8. doi: 10.1016/j.ygyno.2013.05.040 23747291

[6] Armstrong DK, Alvarez RD, Bakkum-Gamez JN, Barroilhet L, Behbakht K, Berchuck A. NCCN Guidelines Insights: Ovarian Cancer, Version 1.2019. J Natl Compr Canc Netw. 2019;17(8):896–909. 31390583 10.6004/jnccn.2019.0039

[7] Jones NL, Chen L, Chatterjee S, Tergas AI, Burke WM, Hou JY, et al. National trends in extended procedures for ovarian cancer debulking surgery. Int J Gynecol Cancer. 2018;28(1):19–25. doi: 10.1097/IGC.0000000000001132 28953134 PMC5734991

[8] Polan RM, Slota JM, Barber EL. Postoperative complications in women with ovarian cancer stratified by cytoreductive surgery outcome. J Surg Oncol. 2023;128(5):891–901. doi: 10.1002/jso.27380 37382209 PMC10529113

[9] Salman L, Covens A, Gien LT, Vicus D. Postoperative complications in elderly patients undergoing surgery for ovarian cancer: a NSQIP analysis. J Surg Oncol. 2025;132(3):473–9. doi: 10.1002/jso.70034 40608006

[10] Vogel TR, Dombrovskiy VY, Carson JL, Graham AM, Lowry SF. Postoperative sepsis in the United States. Ann Surg. 2010;252(6):1065–71. doi: 10.1097/SLA.0b013e3181dcf36e 20571363 PMC2951484

[11] Nagalingam K. Understanding sepsis. Br J Nurs. 2018;27(20):1168–70. doi: 10.12968/bjon.2018.27.20.1168 30418843

[12] Deinhardt-Emmer S, Chousterman BG, Schefold JC, Flohé SB, Skirecki T, Kox M, et al. Sepsis in patients who are immunocompromised: diagnostic challenges and future therapies. Lancet Respir Med. 2025;13(7):623–37. doi: 10.1016/S2213-2600(25)00124-9 40409328

[13] Mirouse A, Vigneron C, Llitjos J-F, Chiche J-D, Mira J-P, Mokart D, et al. Sepsis and cancer: an interplay of friends and foes. Am J Respir Crit Care Med. 2020;202(12):1625–35. doi: 10.1164/rccm.202004-1116TR 32813980

[14] Danai PA, Moss M, Mannino DM, Martin GS. The epidemiology of sepsis in patients with malignancy. Chest. 2006;129(6):1432–40. doi: 10.1378/chest.129.6.1432 16778259

[15] Sakr Y, Elia C, Mascia L, Barberis B, Cardellino S, Livigni S, et al. The influence of gender on the epidemiology of and outcome from severe sepsis. Crit Care. 2013;17(2):R50. doi: 10.1186/cc12570 23506971 PMC3733421

[16] Masoomi H, Blumenauer BJ, Blakkolb CL, Marques ES, Greives MR. Predictors of blood transfusion in autologous breast reconstruction surgery: a retrospective study using the nationwide inpatient sample database. J Plast Reconstr Aesthet Surg. 2019;72(10):1616–22. doi: 10.1016/j.bjps.2019.06.012 31331721

[17] Rim DS, Kim BS, Sharma K, Shin J-H, Kim DW. Prior bariatric surgery and risk of poor in-hospital outcomes in COVID-19: findings from a National Inpatient Sample. Surg Obes Relat Dis. 2023;19(12):1435–43. doi: 10.1016/j.soard.2023.07.006 37612187

[18] Rumalla KC, Chandrupatla SR, Singh JA. Intersectional racial, ethnic, and sex-based disparities in length of stay after total hip and knee arthroplasty: An analysis of national data. Osteoarthritis Cartilage. 2026;34(1):160–6. doi: 10.1016/j.joca.2025.10.016 41175923

[19] Cham S, Chen L, St. Clair CM, Hou JY, Tergas AI, Melamed A, et al. Development and validation of a risk-calculator for adverse perioperative outcomes for women with ovarian cancer. Am J Obst Gynecol. 2019;220(6):571.e1–.e8. 10.1016/j.ajog.2019.02.019 PMC6545148 30771346

[20] Cao X, Liu X, Zhang X, Zhang K, Chen C, Yang Q, et al. Risk factors for perioperative blood transfusion in patients undergoing total laparoscopic hysterectomy. BMC Womens Health. 2024;24(1):65. doi: 10.1186/s12905-024-02908-4 38267957 PMC10809697

[21] Chan GCK, Sun T, Stjepanović D, Vu G, Hall WD, Connor JP, et al. Designing observational studies for credible causal inference in addiction research-Directed acyclic graphs, modified disjunctive cause criterion and target trial emulation. Addiction. 2024;119(6):1125–34. doi: 10.1111/add.16442 38343103

[22] Kim JH. Multicollinearity and misleading statistical results. Korean J Anesthesiol. 2019;72(6):558–69. doi: 10.4097/kja.19087 31304696 PMC6900425

[23] Mansournia MA, Geroldinger A, Greenland S, Heinze G. Separation in logistic regression: causes, consequences, and control. Am J Epidemiol. 2018;187(4):864–70. 29020135 10.1093/aje/kwx299

[24] Greenland S, Mansournia MA, Altman DG. Sparse data bias: a problem hiding in plain sight. BMJ. 2016:i1981. doi: 10.1136/bmj.i1981 27121591

[25] Barnato AE, Alexander SL, Linde-Zwirble WT, Angus DC. Racial variation in the incidence, care, and outcomes of severe sepsis: analysis of population, patient, and hospital characteristics. Am J Respir Crit Care Med. 2008;177(3):279–84. doi: 10.1164/rccm.200703-480OC 17975201 PMC2720103

[26] Mayr FB, Yende S, Linde-Zwirble WT, Peck-Palmer OM, Barnato AE, Weissfeld LA, et al. Infection rate and acute organ dysfunction risk as explanations for racial differences in severe sepsis. JAMA. 2010;303(24):2495–503. doi: 10.1001/jama.2010.851 20571016 PMC3910506

[27] Martin GS, Mannino DM, Eaton S, Moss M. The epidemiology of sepsis in the United States from 1979 through 2000. N Engl J Med. 2003;348(16):1546–54. doi: 10.1056/NEJMoa022139 12700374

[28] Rush B, Wiskar K, Celi LA, Walley KR, Russell JA, McDermid RC, et al. Association of household income level and in-hospital mortality in patients with sepsis: a nationwide retrospective cohort analysis. J Intensive Care Med. 2018;33(10):551–6. doi: 10.1177/0885066617703338 28385107 PMC5680141

[29] Goodwin AJ, Nadig NR, McElligott JT, Simpson KN, Ford DW. Where you live matters: the impact of place of residence on severe sepsis incidence and mortality. Chest. 2016;150(4):829–36. doi: 10.1016/j.chest.2016.07.004 27445093 PMC5812766

[30] Arce D, Lee A. Disparities in obstetric sepsis and strategies to prevent them. Semin Perinatol. 2024;48(7):151979. doi: 10.1016/j.semperi.2024.151979 39307594

[31] Yang Q, Wang J, Xu Y, Chen Y, Lian Q, Zhang Y. Incidence and risk factors of in-hospital prosthesis-related complications following total hip arthroplasty: a retrospective Nationwide Inpatient Sample database study. Int Orthop. 2020;44(11):2243–52. doi: 10.1007/s00264-020-04682-y 32594223

[32] Ahlberg CD, Wallam S, Tirba LA, Itumba SN, Gorman L, Galiatsatos P. Linking Sepsis with chronic arterial hypertension, diabetes mellitus, and socioeconomic factors in the United States: a scoping review. J Crit Care. 2023;77:154324. doi: 10.1016/j.jcrc.2023.154324 37159971

[33] Bronheim RS, Oermann EK, Cho SK, Caridi JM. Coagulation profile as a risk factor for 30-day morbidity following cervical laminectomy and fusion. Spine (Phila Pa 1976). 2018;43(4):239–47. doi: 10.1097/BRS.0000000000002301 28658042

[34] Carey IM, Critchley JA, DeWilde S, Harris T, Hosking FJ, Cook DG. Risk of infection in type 1 and type 2 diabetes compared with the general population: a matched cohort study. Diabetes Care. 2018;41(3):513–21. doi: 10.2337/dc17-2131 29330152

[35] Violi F, Castellani V, Menichelli D, Pignatelli P, Pastori D. Gut barrier dysfunction and endotoxemia in heart failure: a dangerous connubium? Am Heart J. 2023;264:40–8. doi: 10.1016/j.ahj.2023.06.002 37301317

[36] Evans CE, Iruela-Arispe ML, Zhao Y-Y. Mechanisms of endothelial regeneration and vascular repair and their application to regenerative medicine. Am J Pathol. 2021;191(1):52–65. doi: 10.1016/j.ajpath.2020.10.001 33069720 PMC7560161

[37] Zhang L, Zhang F, Xu F, Wang Z, Ren Y, Han D, et al. Construction and evaluation of a sepsis risk prediction model for urinary tract infection. Front Med (Lausanne). 2021;8:671184. doi: 10.3389/fmed.2021.671184 34095176 PMC8175780

[38] Kreimeier U. Pathophysiology of fluid imbalance. Crit Care. 2000;4 Suppl 2(Suppl 2):S3–7. doi: 10.1186/cc968 11255592 PMC3226173

[39] Cryan JF, O’Riordan KJ, Sandhu K, Peterson V, Dinan TG. The gut microbiome in neurological disorders. Lancet Neurol. 2020;19(2):179–94. doi: 10.1016/S1474-4422(19)30356-4 31753762

[40] Adelman MW, Woodworth MH, Langelier C, Busch LM, Kempker JA, Kraft CS, et al. The gut microbiomes role in the development, maintenance, and outcomes of sepsis. Crit Care. 2020;24(1):278. doi: 10.1186/s13054-020-02989-1 32487252 PMC7266132

[41] Angus DC, van der Poll T. Severe sepsis and septic shock. N Engl J Med. 2013;369(9):840–51. doi: 10.1056/NEJMra1208623 23984731

[42] Moore S, Weiss B, Pascual JL, Kaplan LJ. Management of acute respiratory failure in the patient with sepsis or septic shock. Surg Infect (Larchmt). 2018;19(2):191–201. doi: 10.1089/sur.2017.297 29360422

[43] Chenoweth CE. Urinary tract infections. Infect Dis Clin North Am. 2021;35(4):857–70. 34752223 10.1016/j.idc.2021.08.003

[44] Li Z, Shan X, Yang G, Dong L. LGK974 suppresses the formation of deep vein thrombosis in mice with sepsis. Int Immunopharmacol. 2024;127:111458. doi: 10.1016/j.intimp.2023.111458 38160565

[45] Leone M, Nielsen ND, Russell L. Ten tips on sepsis-induced thrombocytopenia. Intensive Care Med. 2024;50(7):1157–60. doi: 10.1007/s00134-024-07478-5 38739278

[46] Claushuis TAM, van Vught LA, Scicluna BP, Wiewel MA, Klein Klouwenberg PMC, Hoogendijk AJ, et al. Thrombocytopenia is associated with a dysregulated host response in critically ill sepsis patients. Blood. 2016;127(24):3062–72. doi: 10.1182/blood-2015-11-680744 26956172

[47] Hansrani V, Khanbhai M, McCollum C. The prevention of venous thromboembolism in surgical patients. Thrombosis and embolism: from research to clinical practice. Advances in Experimental Medicine and Biology. 2016. pp. 1–8. 10.1007/5584_2016_100 27620304

[48] Arfaras-Melainis A, Polyzogopoulou E, Triposkiadis F, Xanthopoulos A, Ikonomidis I, Mebazaa A, et al. Heart failure and sepsis: practical recommendations for the optimal management. Heart Fail Rev. 2020;25(2):183–94. doi: 10.1007/s10741-019-09816-y 31227942

[49] Straubhar AM, Wolf JL, Zhou MQC, Iasonos A, Cham S, Wright JD, et al. Advanced ovarian cancer and cytoreductive surgery: independent validation of a risk-calculator for perioperative adverse events. Gynecol Oncol. 2021;160(2):438–44. doi: 10.1016/j.ygyno.2020.11.021 33272645 PMC7856180

[50] Kumar A, Janco JM, Mariani A, Bakkum-Gamez JN, Langstraat CL, Weaver AL, et al. Risk-prediction model of severe postoperative complications after primary debulking surgery for advanced ovarian cancer. Gynecol Oncol. 2016;140(1):15–21. doi: 10.1016/j.ygyno.2015.10.025 26541980

[51] Liu B, Hadzi-Tosev M, Liu Y, Lucier KJ, Garg A, Li S, et al. Accuracy of international classification of diseases, 10th revision codes for identifying sepsis: a systematic review and meta-analysis. Crit Care Explor. 2022;4(11):e0788. doi: 10.1097/CCE.0000000000000788 36382338 PMC9649267

[52] Quan H, Li B, Saunders LD, Parsons GA, Nilsson CI, Alibhai A, et al. Assessing validity of ICD-9-CM and ICD-10 administrative data in recording clinical conditions in a unique dually coded database. Health Serv Res. 2008;43(4):1424–41. doi: 10.1111/j.1475-6773.2007.00822.x 18756617 PMC2517283

[53] Chute CG, Huff SM, Ferguson JA, Walker JM, Halamka JD. There are important reasons for delaying implementation of the new ICD-10 coding system. Health Aff (Millwood). 2012;31(4):836–42. doi: 10.1377/hlthaff.2011.1258 22442180

