# Supplementary figures and images for: The incidence and risk factors of sepsis following ovarian cancer surgery: A retrospective Nationwide Inpatient Sample database study
Source: PLoS One. 2026 Jul 20;21(7):e0353675. doi: 10.1371/journal.pone.0353675 (PMC13384307; doi:10.1371/journal.pone.0353675)

S1 Fig. Directed Acyclic Graph (DAG)


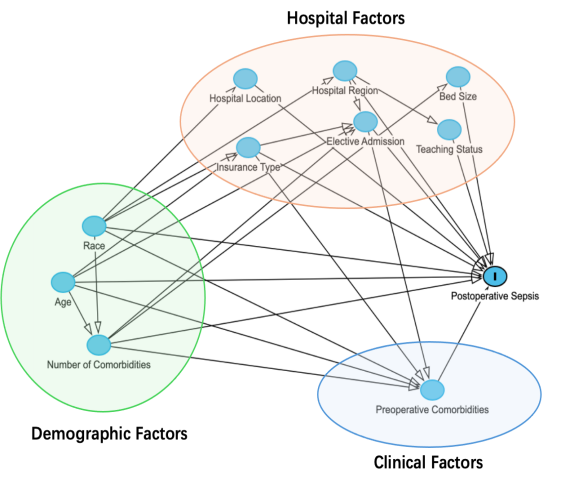

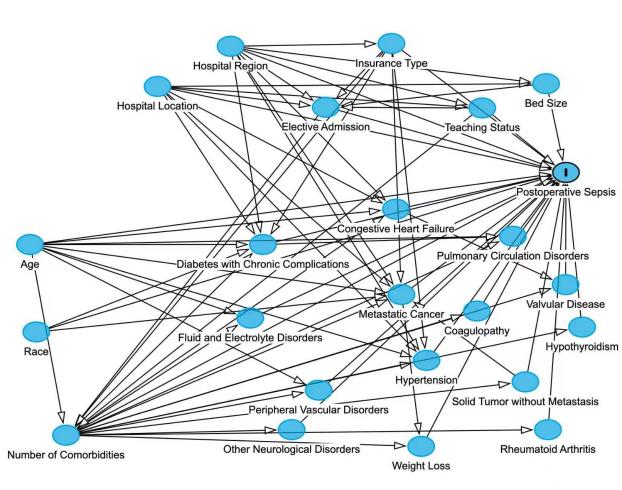

Supplement: S1 Fig — (DOCX) [file pone.0353675.s001.docx]
