# Supplementary material for: The incidence and risk factors of sepsis following ovarian cancer surgery: A retrospective Nationwide Inpatient Sample database study
Source: PLoS One. 2026 Jul 20;21(7):e0353675. doi: 10.1371/journal.pone.0353675 (PMC13384307; doi:10.1371/journal.pone.0353675)
Supplement: S2 Table — NIS: National Inpatient Sample. (DOCX) [file pone.0353675.s003.docx]

S2 Table. Definition of Bed size of hospital in the NIS database

|  | Bed Size of Hospital | | | |
| --- | --- | --- | --- | --- |
| Region of Hospital | Location and Teaching Status  Of Hospital | Bed Size of Hospital (Number) | | |
|  |  | Small | Medium | Large |
| Northeast | Rural | 1-49 | 50-99 | 100+ |
|  | Urban, nonteaching | 1-124 | 125-199 | 200+ |
|  | Urban, teaching | 1-249 | 250-424 | 425+ |
| Midwest | Rural | 1-29 | 30-49 | 50+ |
|  | Urban, nonteaching | 1-74 | 75-174 | 175+ |
|  | Urban, teaching | 1-249 | 250-374 | 375+ |
| South | Rural | 1-39 | 40-74 | 75+ |
|  | Urban, nonteaching | 1-99 | 100-199 | 200+ |
|  | Urban, teaching | 1-249 | 250-449 | 450+ |
| West | Rural | 1-24 | 25-44 | 45+ |
|  | Urban, nonteaching | 1-99 | 100-174 | 175+ |
|  | Urban, teaching | 1-199 | 200-324 | 325+ |

NIS: National Inpatient Sample
