# Supplementary material for: The incidence and risk factors of sepsis following ovarian cancer surgery: A retrospective Nationwide Inpatient Sample database study
Source: PLoS One. 2026 Jul 20;21(7):e0353675. doi: 10.1371/journal.pone.0353675 (PMC13384307; doi:10.1371/journal.pone.0353675)
Supplement: S3 Table — (DOCX) [file pone.0353675.s004.docx]

S3 Table. The ICD-9/10 code of complications

|  | ICD-9 code of complications | ICD-10 code of complications |
| --- | --- | --- |
| Blood transfusion | 30230H1/30230J1/30230K1/30230L1/30230M1/30230N1/30230P1/30230Q1/30230R1/30230S1/30233H1/30233J1/30233K1/30233L1/30233M1/30233N1/30233P1/30233Q1/30233R1/30233S1/30240H1/30240J1/30240K1/30240L1/30240M1/30240N1/30240P1/30240Q1/30240R1/30240S1/30243H1/30243J1/30243K1/30243L1/30243M1/30243N1/30243P1/30243Q1/30243R1/30243S1/30250H1/30250J1/30250K1/30250L1/30250M1/30250N1/30250P1/30250Q1/30250R1/30250S1/30253H1/30253J1/30253K1/30253L1/30253M1/30253N1/30253P1/30253Q1/30253R1/30253S1/30260H1/30260J1/30260K1/30260L1/30260M1/30260N1/30260P1/30260Q1/30260R1/30260S1/30263H1/30263J1/30263K1/30263L1/30263M1/30263N1/30263P1/30263Q1/30263R1/30263S1/30230H0/30230J0/30230K0/30230L0/30230M0/30230N0/30230P0/30230Q0/30230R0/30230S0/30233H0/30233J0/30233K0/30233L0/30233M0/30233N0/30233P0/30233Q0/30233R0/30233S0/30240H0/30240J0/30240K0/30240L0/30240M0/30240N0/30240P0/30240Q0/30240R0/30240S0/30243H0/30243J0/30243K0/30243L0/30243M0/30243N0/30243P0/30243Q0/30243R0/30243S0/30250H0/30250J0/30250K0/30250L0/30250M0/30250N0/30250P0/30250Q0/30250R0/30250S0/30253H0/30253J0/30253K0/30253L0/30253M0/30253N0/30253P0/30253Q0/30253R0/30253S0/30260H0/30260J0/30260K0/30260L0/30260M0/30260N0/30260P0/30260Q0/30260R0/30260S0/30263H0/30263J0/30263K0/30263L0/30263M0/30263N0/30263P0/30263Q0/30263R0/30263S0 | 9900/9902/9903/9904/9905/9907/9908/ V582 |

continue

| Urinary tract infection (UTI) | N390/N3000/N3001/N3030/N3031/N3080/N3081/N3090/N3091/N340/N341/N342/N343/T83021A/T83021D/T83021S | 590/5901/5902/5903/5909/595/5950/5953/5954/5958/5959/5970/5978/5990/ 9975 |
| --- | --- | --- |
| Thrombocytopenia | D6951/D6959/D696 | 2874/2875 |
| Respiratory disease | J9500/J9501/J9502/J9503/J9504/J9509/J952/J953/J9561/J9562/J9571/J9572/J95811/J95812/J95830/J95831/J95850/J95851/J95859/J95860/J95861/J95862/J95863/J9588/J9589/T8182XA/T8182XD/T8182XS | 99739/51900/51901/51902/51909/51852/51882/V4614/86120/5187/99881/5122/5198/5199/51919 |
| Genitourinary disease | N000/N001/N002/N003/N004/N005/N007/N008/N009/N040/N041/N042/N043/N044/N045/N047/N048/N049/N10/N170/N171/N172/N178/N179/N250/N251/N2581/N2589/N259/N3000/N3001/N3030/N3031/N3080/N3081/N3090/N3091/N340/N341/N342/N343/N990/N991/N9981/N99821 | 5800/5804/58081/58089/5809/5810/5811/5812/5813/58181/58189/5819/5845/5846/5847/5848/5849/5880/5881/58881/58889/5889/59000/59001/59010/59011/5902/5903/59081/59080/5909/595/5950/5952/5953/5954/59581/59582/599589//5959/5970/5978/59781/59789/5990/9975/5999/59389/5939/59653/5966/5968/5969 |
| Pneumonia | J120/J121/J122/J123/J1281/J1289/J129/J13/J14/J150/J151/J1520/J15211/J15212/J1529/J153/J154/J155/J156/J157/J158/J159/J160/J168/J17/J180/J181/J182/J188/J189/J678/J679/J954 | 4800/4801/4802/4803/4808/4809/481/4820/4821/4822/48230/48231/48232/48239/48240/48241/48242/48249/48281/48282/48283/48284/48289/4829/4830/4831/4838/4841/4843/4845/4846/4847/4848/485/486/4870/5070/5071/5078/51630/51632/51633/51635/51636/51637/99731/99732 |

continue

| Gastrointestinal complication | K910/K911/K912/K9130/K9131/K9132/K9172/K91841/K91850/K91858/K91871/K91873/K9189 | 9974 |
| --- | --- | --- |
| Deep venous thrombosis (DVT) | I82210/I82220/I82290/I823/I82401/I82402/I82403/I82409/I82411/I82412/I82413/I82419/I82421/I82422/I82423/I82429/I82431/I82432/I82433/I82439/I82441/I82442/I82443/I82449/I82451/I82452/I82453/I82459/I82461/I82462/I82463/I82469/I82491/I82492/I82493/I82499/I82621/I82622/I82623/I82629 | 4510/45111/45119/4512/4518/45181/45182/45183/45184/45189/4519/45340/45341/45342/4538/45381/45382/45383/45384/45385/45386/45387/45389 |
| Wound infection | T8140/T8141/T8142/T8143/T8149/T814XXA、T814XXD、T814XXS | 99851/99859/99883 |
| Pulmonary embolism (PE) | I2601/I2602/I2609/I2690/I2692/I2693/I2694/I2699 | 41511/41512/41513/41519/ |
| urinary retention | R338，R339 | 78820/78821/78829 |
| respiratory failure | J95821/J95822/J9610/J9611/J9612/J9620/J9621/J9622/J9690/J9691/J9692 | 51851/51852/51853/51883/51884 |
| Continuous trauma ventilation | 9670/9671/9672 | F028GCZ/F028GGZ/F028GYZ/F028GZZ/5A09357/5A09358/5A0935B/5A09359/5A0935Z/5A09457/5A09458/5A09459/5A0945B/5A0945Z/5A09557/5A09558/5A09559/5A0955B/5A0955Z |
| Heart failure | I501/I5020/I5021/I5030/I5031/I5040/I5041/I5043/I50810/I50811/I50814/I5082/I5083/I5084/I5089/I509 | 4280/4281/42820/42821/42830/42831/42840/42841/42843/4289 |
